# Cardiovascular risk factors and cognitive function in middle aged and elderly Lithuanian urban population: results from the HAPIEE study

**DOI:** 10.1186/1471-2377-12-149

**Published:** 2012-11-30

**Authors:** Abdonas Tamosiunas, Migle Baceviciene, Regina Reklaitiene, Ricardas Radisauskas, Kristina Jureniene, Adelina Azaraviciene, Dalia Luksiene, Vilija Malinauskiene, Evelina Daugeliene, Laura Sapranaviciute-Zabazlajeva

**Affiliations:** 1 Lithuanian University of Health Sciences, Academy of Medicine, Institute of Cardiology, Sukileliu 17, 50009, Kaunas, Lithuania

## Abstract

**Background:**

The purpose of this study was to examine associations between cardiovascular risk factors and cognitive ability in middle aged and elderly Lithuanian urban population.

**Methods:**

Data from the survey performed in the framework of the HAPIEE (Health, Alcohol, Psychosocial Factors in Eastern Europe) study were presented. A random sample of 7,087 individuals aged 45–72 years was screened in 2006–2008.

**Results:**

The scores of immediate recall and delayed verbal recall, cognitive speed and attention were significantly lower in men than in women; yet numerical ability scores were higher in men. Significant associations between lowered cognitive functions and previous stroke (in male OR = 2.52; 95% CI = 1.75-3.64; in female OR = 2.45; 95% CI = 1.75, 3.64) as well as ischemic heart disease history (among male OR = 1.28; 95% CI = 1.03-1.60) have been determined. Higher level of physical activity in leisure time (among female OR = 1.32; 95% CI = 1.03-1.69), poor self-rated health (among male OR = 1.57; 95% CI = 1.15-2.14) and poor quality of life (in male OR = 1.67; 95% CI = 1.07-2.61; in female OR = 2.81; 95% CI = 1.92-4.11) were related to lowered cognitive function.

**Conclusions:**

The findings of the study suggest that associations between cardiovascular risk factors and lowered cognitive function among healthy middle-aged and elderly adults strongly depend on gender.

## Background

The ageing of populations worldwide raises concern regarding the prevalence of cognitive dysfunction in the future. Cognitive decline and dementia have a high individual impact and are strongly age-associated. A consensus report estimated that the number of people with dementia in the world will increase from 24 million to 82 million from 2000 to 2040 [1]. This increase will be particularly marked in low and middle income countries where epidemiological research into the etiology and impact of cognitive impairment is limited. Therefore, it is becoming increasingly important to identify risk factors associated with cognitive functions to better target the preventive strategies and public health messages.

Cardiovascular disease (CVD) and cognitive decline are common problems in middle aged and elderly men and women [2]. Epidemiological studies have linked cognitive performance with CVD, specific vascular biomarkers and risk factors such as cholesterol, hypertension, diabetes mellitus, fibrinogen levels, homocysteine, C-reactive protein [3-5] and lifestyle behavior [6,7]. However, the associations are not well established – researchers have yielded mixed findings. In accordance with literature sources and given the high levels of mortality from CVD and unhealthy lifestyles in Lithuania [8-11] we hypothesized that lowered cognitive functions could be associated with CVD and its risk factors. The aim of this study, therefore, was to explore associations between cognitive ability, CVD and its risk factors in middle aged and elderly Lithuanian urban population.

## Methods

### Study design

Data from the survey performed in the framework of the international HAPIEE (Health, Alcohol and Psychosocial Factors in Eastern Europe) study are presented [12]. A random sample of 10,940 Kaunas (Lithuania) men and women aged 45–72 years, stratified by gender and age was selected from Lithuanian population register. The response rate was 64.8%, thus 7,087 respondents participated in this health survey from 2006 to 2008. The participants were asked at invitation to bring their glasses and hearing aids, the absence of it was noted. The subjects who were reported that vision/ hearing problems interfered with their tests were excluded from the analysis the same as the individuals who refused to finish some tests. In total 183 responders were excluded from the statistical analysis because of incomplete information. Data from 6,904 subjects (3,129 men and 3,775 women) were approved for statistical analysis. The study was approved by the Ethics Committee at University College London, UK and by Kaunas Regional Biomedical Research Ethics Committee.

### Cognitive function

For the purpose of controlling conditions of cognitive testing, it was executed in a separate consulting room in the morning. The procedure was administered by specially trained staff [12]. Cognitive function was assessed using a battery of five standard tasks, described below. These tests of cognitive function have been taken from the English Longitudinal Study of Ageing (ELSA) and Consortium to Establish a Registry for Alzheimer’s Disease (CERAD) study [13,14]. *Immediate and delayed verbal memory* was assessed using 10-word learning test. This involved 10 common two-to-four syllable nouns being presented aurally by tape recorder at the rate of one word every 2 seconds. The participants were then asked to recall as many words as possible immediately and they had two minutes to do so. The test was repeated three times using the same procedure. The maximum score of any trial is 10 (range: 0–10). The cumulative total maximum score over all three learning trials was 30 (range: 0–30). The participants were also asked to recall as many words as possible again after an approximately a five-minute delay during which they completed other cognitive function tests. The maximum score of delayed verbal memory is 10 (range: 0–10). *Semantic verbal fluency* was examined by asking the participants to name as many animals as possible within 1 minute. *Speed and concentration* were tested by asking the participants to cross out as many target letters (‘P’ and ‘W’) as possible within 1 minute, using a sheet with random letters of the alphabet set out in rows and columns. Numerical ability was assessed using four questions involving simple calculations based on everyday situations. The number of correct responses comprised the numeracy score (in the range 0 to 4).

#### Composite score of cognitive function

Because the scoring of each participant cognitive tests varies, test scores were standardized to give a mean of 0 and a standard deviation of 1 (z-scores), where high scores represent high levels of cognitive function. Scores representing a composite score of cognitive function were obtained by averaging z-scores in all tests.

#### Lowered cognitive function

Lowered cognitive function was defined using composite score of cognitive function. To control for the effect of age and education on scores, the subjects were stratified into six age groups and five levels of education. For each of 30 strata, the mean and SD of the composite score of cognitive function was calculated. The participants who scored 1 SD or more below their age and education-specific means of the composite score of cognitive function were ascribed to a lowered cognitive function group [15,16].

### Variables determined using the questionnaire

The standard questionnaire included questions regarding the respondent’s age, education, self-rated health, quality of life, smoking status, alcohol consumption, physical activity, and etc. Education was classified into five education levels: primary, vocational, secondary, college and university. Marital status of all study participants was divided into five groups: single, married, cohabited, divorced and widowed. An indicator of material deprivation was assessed by questions about how often the person’s household had difficulties in buying enough food or clothes and paying bills for housing, heating and electricity. A higher deprivation score means a lower level of deprivation. Assessment of crowding was based on the number of inhabitants living in a household. The respondents were categorized into the following groups according to their self-rated health and quality of life: very good, good, average, poor, and very poor.

Smoking habits were assessed according to the current smoking status. The respondents were classified into three groups: smokers, former smokers and never smokers. A subject who smoked at least one cigarette per day was classified as current smoker. Alcohol consumption was measured by asking participants how often they drank 5 and more alcoholic beverages and how often they drank less than 5 drinks during the year. Additionally, participants were asked to write how many deciliters of different alcoholic beverages (beer, wine, and spirits) they drank per week. Alcohol consumption was expressed by amount of absolute alcohol drinks per week. Physical activity was determined by meantime spent per week during leisure time in winter and summer for walking, moderate and hard work, gardening and other physical activities. The respondents were categorized into three groups according to their physical activity in leisure time: in men, 1^st^ tercile (0.0-10.5 hours/week), 2^nd^ tercile (10.61-20.0 hours/week), and 3^rd^ tercile (> 20.0 hours/week); in women, 1^st^ tercile (0.0-14.0 hours/week), 2^nd^ tercile (14.1-22.5 hours/week), and 3^rd^ tercile (> 22.5 hours/week).

Depressive symptoms were measured using the 10-item Center for Epidemiologic Studies Depression Scale (CES-D 10) [17]. Specially trained personnel filled in questionnaires by asking interviewing the respondents. The subjects were asked to evaluate the presence of 10 depressive symptoms during the past week on a two-point scale: yes or no. Each symptom was scored from 1 (yes) to 0 (no), resulting total score of 0 to 10. The subjects with CES-D 10 scores of 4 or more were classified as having depressive symptoms.

### Measurements

Blood pressure was measured three times, using an oscillometric device (Omron M5-I) after a five minutes’ rest. The mean of three systolic and diastolic blood pressure was used. Waist circumference was measured by a standard meter with an accuracy of 0.5 cm. Body mass index (BMI) was calculated as weight (kg) divided by the square meters of height.

### Laboratory analyses

Biochemical analyses were conducted for participants on an empty stomach. Concentration of glucose in capillary blood was determined by an individual glucometer “Glucotrend” [18]. Serum samples were analyzed centrally in one batch in the WHO Regional Lipid Reference Centre, Institute of Clinical and Experimental Medicine, Prague (Czech Republic). Lipid concentrations in serum were measured, using a conventional enzymatic method.

### Definitions of the health conditions

*Hypertension* was defined as systolic blood pressure ≥ 140 mm Hg and/or diastolic blood pressure ≥ 90 mm Hg, or normal blood pressure (< 140/90 mm Hg) if the person had taken antihypertensive drugs within the last two weeks. *Metabolic syndrome* was defined as the presence of three or more of five components: systolic/diastolic blood pressure ≥130 and/or 85 mmHg; fasting plasma glucose ≥6.1 mmol/L; central obesity (waist circumference >102 cm for men and >88 cm for women); fasting triglycerides ≥1.7 mmol/L; high density lipoprotein (HDL) cholesterol <1.0 mmol/L for men and <1.3 mmol/L for women [19]. *Ischemic heart disease (IHD)* was determined by: 1) documented history of myocardial infarction (MI) and (or) ischemic changes on ECG coded by the Minnesota codes (MC) 1–1 or 1–2 [20]; 2) angina pectoris was defined by G. Rose questionnaire (without MI and (or) MC 1–1 or 1–2; 3) [21]; ECG findings by MC 1–3, 4–1, 4–2, 4–3, 5–1, 5–2, 5–3, 6–1, 6–2, 7–1, 8–3 (without MI and (or) MC 1–1, 1–2 and without angina pectoris). *Diabetes* was determined according to the answers of the respondents to the question “Has a doctor ever told you that you have diabetes?” and/or fasting glucose level ≥7.8 mmol/L. *Stroke* was determined using the question “Has a doctor ever told you that you had a stroke?”.

### Statistical analysis

Descriptive statistics (unadjusted and adjusted by age means and standard errors (SE)) were calculated for each of the cognitive function tests in gender, age and education groups. In separate analyses, we examined age-adjusted means and distribution of various variables according to the cognitive function in gender groups. Chi-square (χ^2^) tests were used for testing association of various variables with cognitive function (lowered cognitive function vs. normal cognitive function) in gender groups. Differences in continuous measures between categories of cognitive function were tested via t-test. Multiple logistic regression with SPSS version 13.4 software for windows was used to analyze risk factors associated with a lowered cognitive function [22]. All independent variables which were significant in univariate analysis with the significance level 0.95 (p < 0.05) were then tested by stepwise backward multiple logistic regression analysis using the likelihood ratio criterion.

## Results

The crude and age-adjusted mean scores of specific cognitive function tests and composite scores of cognitive function for men and women are presented in Table 1. It is apparent that, unadjusted and age-adjusted means of immediate and delayed verbal recall, cognitive speed and attention, composite score of cognitive function were significantly lower in men than in women. Semantic verbal fluency score was similar in both gender groups. The mean score of numerical ability was significantly higher among men as compared to women.

**Table 1:** Cognitive function in Kaunas population by sex, age 45–72 years

**Cognitive function test**	**Unadjusted**		**Age-adjusted**	
	**Men N = 3,129**	**Women N = 3,775**	**Men N = 3,129**	**Women N = 3,775**
	**Mean ± SE**	**Mean ± SE**	**Mean ± SE**	**Mean ± SE**
Immediate verbal recall I	5.45 ± 0.03	6.02 ± 0.03***	5.46 ± 0.03	6.01 ± 0.02***
Immediate verbal recall II	7.26 ± 0.03	7.90 ± 0.02***	7.27 ± 0.03	7.89 ± 0.02***
Immediate verbal recall III	8.13 ± 0.03	8.69 ± 0.02***	8.14 ± 0.03	8.67 ± 0.02***
Immediate verbal recall sum	20.8 ± 0.07	22.6 ± 0.06***	20.9 ± 0.07	22.6 ± 0.06***
Delayed verbal recall	7.32 ± 0.04	8.02 ± 0.03***	7.33 ± 0.03	8.01 ± 0.03***
Semantic verbal fluency	21.5 ± 0.11	21.4 ± 0.10	21.5 ± 0.11	21.4 ± 0.10
Numerical ability	3.07 ± 0.01	2.85 ± 0.01***	3.07 ± 0.01	2.85 ± 0.01***
Cognitive speed and attention	15.4 ± 0.08	16.8 ± 0.08***	15.5 ± 0.08	16.73 ± 0.07***
Composite score of cognitive function	−0.12 ± 0.12	0.1 ± 0.01***	−0.12 ± 0.01	0.1 ± 0.01***

SE - standard error.

***p < 0.001 as compared to men (means were compared using t test).

In additional analyses it was found that the means of all cognitive functions significantly decreased with age and, in opposite, they increased with higher education level among both men and women (p < 0.001).

The prevalence of lowered cognitive function was 20.6% among men and 11.8% among women (p < 0.001). In both sexes, distribution into groups by cognitive functioning using a composite score as a measure was not related to age and education due to control for both factors. Table 2 reports age-adjusted means and distribution of variables in cognitive function groups by gender.

**Table 2:** **Age-adjusted means (± SE)**^**a**^**and distribution of variables (%) according to the cognitive function**

**Variables**	**Men (n = 3,129)**		**Women (n = 3,775)**	
	**Cognitive function**		**Cognitive function**	
	**Lowered (n = 645)**	**Normal (n = 2,484)**	**Lowered (n = 444)**	**Normal (n = 3,331)**
Age, years	61.0 ± 0.3	60.4 ± 0.15	60.4 ± 0.35	60.3 ± 0.13
Deprivation^a^, score	4.76 ± 0.02	4.79 ± 0.01	4.51 ± 0.03	4.59 ± 0.01*
Crowding^a^, absolute number	2.69 ± 0.05	2.69 ± 0.02	2.35 ± 0.06	2.36 ± 0.02
Systolic blood pressure^a^, mm Hg	148.3 ± 0.84	146.1 ± 0.43*	138.1 ± 0.97	137.2 ± 0.35
Diastolic blood pressure^a^, mm Hg	93.8 ± 0.5	92.7 ± 0.25*	88.7 ± 0.56	88.3 ± 0.2
Total cholesterol^a^, mmol/L	5.73 ± 0.02	5.78 ± 0.02	6.07 ± 0.05	6.13 ± 0.02
HDL cholesterol^a^, mmol/L	1.39 ± 0.02	1.4 ± 0.01	1.52 ± 0.02	1.58 ± 0.01**
LDL cholesterol^a^, mmol/L	3.65 ± 0.04	3.71 ± 0.02	3.86 ± 0.05	3.89 ± 0.02
Triglyceride^a^, mmolL	1.53 ± 0.04	1.47 ± 0.02	1.54 ± 0.04	1.45 ± 0.02*
Fasting blood glucose^a^, mmol/L	5.77 ± 0.05	5.84 ± 0.03	5.94 ± 0.06	5.84 ± 0.02
Body mass index^a^, kg/m^2^	28.6 ± 0.18	28.5 ± 0.09	30.2 ± 0.27	30.1 ± 0.10
Physical activity in leisure time^a^, hours/week	16.4 ± 0.49	17.7 ± 0.25*	18.1 ± 0.56	20.1 ± 0.2*
Absolute alcohol ^a^, drinks/week	57.6 ± 4.6	56.6 ± 2.31	14.6 ± 1.41	17.8 ± 0.51*
Ischemic heart disease (%)	χ^2^ = 12.7; p < 0.001		χ^2^ = 0.21; p > 0.05	
Yes	22.9	16.9	20.5	19.6
No	77.1	83.1	79.5	80.4
Stroke (%)	χ^2^ = 42.1; p < 0.001		χ^2^ = 26.5; p < 0.001	
Yes	9.0	3.1	8.2	3.2
No	91.0	96.9	91.8	99.8
Diabetes mellitus (%)	χ^2^ = 0.02; p > 0.05		χ^2^ = 0.61, p > 0.05	
Yes	8.8	8.6	10.4	9.2
No	91.2	91.4	89.6	90.8
Metabolic syndrome (%)	χ^2^ = 0.00; p > 0.05		χ^2^ = 3.5; p > 0.05	
Yes	29.3	29.4	43.2	38.5
No	70.7	70.6	56.8	61.5
Arterial hypertension (%)	χ^2^ = 0.69; p > 0.05		χ^2^ = 0.83; p > 0.05	
Yes	75.3	73.7	56.8	61.5
No	24.7	26.3	43.2	38.5
Body mass index, (%)	χ^2^ = 3.44; p > 0.05		χ^2^ = 3.92; p > 0.05	
<18.5 kg/m^2^	0.5	0.2	0.5	0.3
18.5- 24.99 kg/m^2^	21.4	21.9	18.7	19.0
25.0-29.9 kg/m^2^	41.7	44.6	31.5	35.7
> = 30.0 kg/m^2^	36.4	33.3	49.3	45.0
Smoking habits (%)	χ^2^ = 0.33; p > 0.05		χ^2^ = 0.71; p > 0.05	
Smokers	30.4	30.6	11.0	9.7
Former smokers	30.1	31.1	7.1	7.3
Never smokers	39.5	38.3	81.9	83.0
Marital status (%)	χ^2^ = 8.32; p > 0.05		χ^2^ = 5.86; p > 0.05	
Single	1.4	2.0	7.3	5.4
Married	80.7	84.3	52.2	56.9
Cohabiting	2.4	1.4	0.5	0.9
Divorced	9.2	7.2	17.6	15.9
Widowed	6.3	5.1	22.4	20.9
Quality of life (%)	χ^2^ = 14.4; p < 0.01		χ^2^ = 37.3; p < 0.001	
Very poor and poor	5.5	2.9	10.4	4.0
Average	49.2	47.1	51.1	50.2
Good	43.1	48.2	37.1	44.5
Very good	2.2	1.8	1.4	1.3
Self-rated health (%)	χ^2^ = 23.6; p < 0.001		χ^2^ = 17.6; p < 0.001	
Very good and good	26.8	34.4	16.9	21.5
Average	57.5	55.6	58.7	61.8
Poor and very poor	15.7	10.0	24.4	16.7
Depression scale score (%)	χ^2^ = 7.28; p < 0.01		χ^2^ = 12.8; p < 0.001	
> = 4	18.9	14.5	37.0	28.7
<4	81.1	85.5	63.0	71.3

^a^ age-adjusted means and standard errors.

SE – standard error.

HDL – high density lipoprotein.

LDL – low density lipoprotein.

* p < 0.05; ** p < 0.01; as compared to lowered cognitive function group (means were compared using t test).

Many differences were found between cardiovascular risk factors and lowered cognitive function due to gender. In men, the means of systolic and diastolic blood pressure were significantly higher in lowered cognitive function group than in normal cognitive function group, yet for women these differences were not significant. Women with lowered cognitive function had significantly higher mean of triglycerides and lower mean of HDL cholesterol, as compared to women with normal cognitive function. There were no significant differences in men scores though. In both sexes LDL cholesterol, total cholesterol and glucose levels did not differ in lowered and normal cognitive function groups. Alcohol intake per week was lower in impaired cognitive function group for women, but not for men. For both sexes smoking status and body mass index did not differ by cognitive function groups. However, the mean time spent for physical activity per week was significantly higher in normal cognitive function group compared to lowered cognitive function group among both sexes. Socioeconomic factors such as crowding and marital status did not significantly differ in cognitive functioning groups, yet deprivation score was lower in lowered cognitive functioning group, which means that higher deprivation level has a group of lowered cognitive function as compared with people who better performed during cognitive functioning tests.

As expected, the distribution of quality of life and self-rated health was significantly related to cognitive function in both sexes. The proportion of the subjects who rated their quality of life and self-rated health as good or very good was lower in the group with lowered cognitive function than in the group with normal cognitive function. Other health conditions are differently related to cognitive functioning. The subjects with lowered cognitive function reported an increased frequency of previous stroke as compared to the subjects with normal cognitive function. The prevalence of IHD was significantly higher in men with lowered cognitive function than in men with normal cognitive function, yet the difference in women group was not significant enough. Diabetes, metabolic syndrome and hypertension were not related to cognitive functioning. People who better performed during cognitive function tests had less depressive symptoms than people with poorer cognitive functions.

Further, variables significantly related to cognitive functioning in univariate analysis were put into the multivariable analysis. Table 3 shows the multivariable odds ratios (ORs) for lowered cognitive function. In both sexes, individuals with previous stroke had significantly higher odds of lowered cognitive function compared to those without stroke: 2.52 times higher among men and 2.45 times higher among women. In men, the individuals with IHD had higher ORs of lowered cognitive function as compared to the men without IHD. The individuals who rated their quality of life as very poor and poor were more likely to demonstrate lowered cognitive performance compared to those who rated their quality of life as very good and good. The men who rated their health as good and very good were more likely to have better cognitive functions than the men who thought that their health was very poor or poor. This association was not significant for women though. For women, physical inactivity in leisure time was related with higher ORs of lowered cognitive function.

**Table 3:** Multivariable adjusted odds ratios (ORs) and 95% confidence intervals (CI) for lowered cognitive function*

**Variables**	**Men**	**Women**
	**OR (95% CI)**	**OR (95% CI)**
Stroke	2.52 (1.75-3.64)	2.45 (1.64-3.66)
Ischemic heart disease	1.28 (1.03-1.60)	n.s.
Quality of life		
Very good and good	1.0	1.0
Neither poor nor good	1.03 (0.86-1.24)	1.16 (0.94-1.44)
Very poor and poor	1.67 (1.07-2.61)	2.81 (1.92-4.11)
Self-rated health		
Good and very good	1.0	n.s.
Average	1.21 (0.99-1.50)	
Poor and very poor	1.57 (1.15-2.14)	
Diastolic blood pressure, mm Hg	1.01 (1.00-1.01)	n.s.
Physical activity in leisure time		
3rd tertile (> 22.5 hours/week)		1.0
2nd tertile (14.1-22.5 hours/week)	n.s.	1.14 (0.88-1.49)
1st tertile (0.0-14.0 hours/week)		1.32 (1.03-1.69)

*- 1 SD or more below age and education specific mean of the composite score of cognitive function.

n.s. – not significant.

In men, ORs are adjusted for previous stroke, ischemic heart disease, quality of life, self-rated health, diastolic blood pressure, physical activity in leisure time, and depression scale score.

In women, ORs are adjusted for previous stroke, quality of life, self-rated health, HDL cholesterol, triglycerides, physical activity in leisure time, alcohol intake, and depression scale score.

Variable selection was conducted by a stepwise backward procedure using the likelihood ratio criterion.

## Discussion

To the best of our knowledge, the presented results are the first estimates of the cognitive function and its associations with various cardiovascular risk factors in a large group of general middle-aged and elderly urban population in Lithuania that were obtained using internationally accepted battery of five standard tasks. This study confirmed the association between older age, lower education level and poorer cognitive performance which had been proposed by previous studies [23-26]. Some gender differences were ascertained as well. In general, women performed marginally better than men on the global cognitive score, conversely to other studies in such countries as Taiwan, Egypt, India or China [25-27]. These results can differ because of high educational level of Lithuanian women. In our study, women performed better on immediate and delayed verbal recall than men. These findings are similar to those of the previous large longitudinal study carried out in Netherlands [28]. Besides, most studies confirm that women have advantages in memory tasks compared to men [25-27,29].

The results of this study indicate that links between cardiovascular risk factors and cognitive function are mixed and highly dependent on gender. Stroke was an independent predictor for lowered cognitive function. This finding supports the results published in by other studies [23,24,26,27]. The previous study suggests association between coronary heart disease history and poorer cognitive scores [3]. In the current study IHD also showed a statistically significant association with lowered cognitive function but among men only. This finding could be explained by higher rates of cardiovascular disease among men in Lithuania [9,10]. It was suggested that cardiovascular risk factors and diabetes, may also be linked with lowered cognitive function [26]. But the current research similarly to some other studies found no consistent associations [24,30]. Metabolic syndrome was not related to poorer cognitive function as well. Researchers in other studies came to a conclusion that association between cognition, metabolic syndrome and its components depends on the specific areas of cognition, which were not analyzed in this study [30,31].

In contrast to the research which reported that hypertension was associated with decreased cognitive function and blood pressure was not [31], we found opposite results. In general, the previous findings suggest very uncertain connections between hypertension and cognition [23,26,31]. Biomarkers were differently associated with lowered cognitive function. Moreover, these associations were confirmed only in univariate analyses. Which biomarkers are related to cognition remains a controversial issue [23,30,31]. Surprisingly, risk behaviors such as smoking and alcohol drinking were not related to lowered cognition differently from the findings in the previous studies [10,11]. However, physical activity was associated with better cognitive performance among women, but not among men.

It had been argued that depressive symptoms regulate association between cognitive functioning and cardiovascular risk factors [2], yet our results indicated that the relation between depressive symptoms and lowered cognitive function was confirmed only in univariate analysis. The implication is that cardiovascular risk factors affect cognition independently. Besides, self-rated quality of life in elderly is influenced by a poorer cognitive function [32,33]. The current investigation revealed that a poor quality of life was the most important predictor of a lowered cognitive function.

Limitations of our findings include the absence of additional cognitive measures such as MMSE which allowed us determine the prevalence of an impaired cognitive function using the standard criteria and compare the prevalence in other populations. Dementia status was not controlled in our research as well. Other limitations of our study include assessment of cognitive function by random sample in Kaunas population, but not on a nationally based measure. Additionally, we used a cross-sectional design despite the HAPIEE study is planned as cohort study. Although incremental models were not analyzed in this research, interactions of possible confounders will be discussed in future studies.

Despite some limitations of the study it is the first population based study in Lithuania which analyzed associations between cognitive ability and various CVD risk factors. This study reflects on biopsychosocial model of health by analyzing cognitive functions in respect to many different factors including clinical data as biomarkers, self-report measurements as depressive symptoms and socio-demographic data.

## Conclusions

This study adds to evidence that the prevalence of lowered cognitive function was significantly higher among Lithuanian middle-aged and elderly urban men as compared to women (20.6% and 11.8%). Previous stroke (in both sexes), IHD and self-rated health (among men only), were significantly associated with odds of lowered cognitive function. Furthermore, the results from this study indicate that physical activity in leisure time (among women) and quality of life (in both sexes) are related with cognitive performance.

## Abbreviations

BMI: Body mass index; ECG: Electrocardiogram; HDL: High density lipoprotein; IHD: Ischemic heart disease; LDL: Low density lipoprotein; MI: Myocardial infarction; MC: Minnesota codes; OR_S_: Odds ratios; SE: Standard error; SD: Standard deviation; WHO: World Health Organization; CVD: Cardiovascular disease.

## Competing interests

The authors declare that they have no competing interests.

## Authors’ contributions

AT contributed to writing the manuscript, the study concept and design and the analysis and interpretation of data. MB contributed to writing the manuscript and the analysis and interpretation of data. RR contributed to the study concept and design and the analysis and interpretation of data. RR, KJ, AA, DL, and VM contributed to writing the manuscript. ED contributed to writing the manuscript, the study concept and design and the analysis and interpretation of data. LS-Z contributed to writing the manuscript and interpretation of data. All authors read and approved the final manuscript.

## Pre-publication history

The pre-publication history for this paper can be accessed here:

http://www.biomedcentral.com/1471-2377/12/149/prepub
